# Temporal changes in diastolic function measured by volumetric CMR after ST Elevation Myocardial infarction

**DOI:** 10.1186/1532-429X-13-S1-P138

**Published:** 2011-02-02

**Authors:** Gopal Ghimire, Jyotshana Shrestha, Manuel A Gonzalez, Ana Barac, Rebecca Torguson, William O Suddath, Lowell F Satler, Augusto D Pichard, Ron Waksman, Anthon R Fuisz, Gaby Weissman

**Affiliations:** 1 Washington Hospital Center, Washington, DC, USA

## Objective

We aim to evaluate changes in diastolic function after ST elevation myocardial infarction (STEMI) using volumetric cardiac magnetic resonance imaging (CMR), as well as the relation of diastolic parameters to baseline variables including peak troponin-I and baseline left ventricular ejection fraction (LVEF).

## Background

Diastolic dysfunction is one of the earliest manifestations of cardiac ischemia. However, the response to reperfusion of diastolic filling parameters in patients with STEMI undergoing primary percutaneous coronary intervention (PCI) is not well elucidated. Using volumetric analysis we aimed to define the temporal change in diastolic function after primary PCI.

## Method

23 patients with STEMI undergoing primary PCI were prospectively enrolled and sequential CMR imaging was performed within 96 hours of admission and at 1 month follow up. LV volumetric quantification was performed (QMass MR, Medis, Leiden, The Netherlands) with manual planimetry of the endocardial contours in all temporal phases of the contiguous short-axis slices. Systolic parameters were obtained. Diastolic CMR parameters were derived including peak filling rate (PFR) and its index normalized to end diastolic volume (NPFR), time to peak filling rate (TPFR), diastolic volume recovery (DVR_80_): proportion of diastole required for recovery of 80 percentage of stroke volume as well as the ratio of the biphasic early (E) and late (A) filling profiles (E/A ratio). The change in those parameters was then correlated to peak troponin-I and baseline LVEF.

## Results

**Figure 1:**
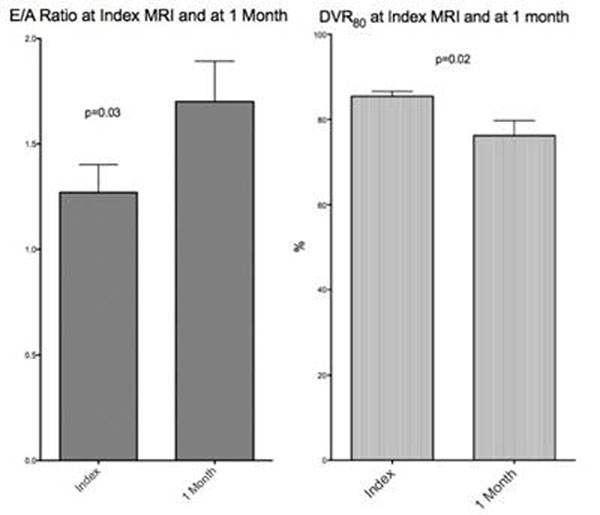


At one month follow-up LVEF improved from 49% (interquartile range (38-61) at baseline to 57% (49-62), p=0.04. There were no significant changes in LV volumes; LV end diastolic volume changed from 194.5ml ( 152.9-237.0) to 192ml (152.5-221.7), p=0.14) and LV end systolic volume from 100ml (65.0-125.8) to 87.71ml (52.8-96.0), p=0.08. The changes in the diastolic parameters are depicted in table 1. PFR, TPFR and NPFR did not change significantly however E/A increased from 1.06 to 1.63 while DVR_80_ decreased from 86.1% to 83.2%. Figure 1.

**Table 1:** 

Diastolic parameters	Index MRI	Follow up MRI	
DVR_8_0	86.1 (91.53-90.12)	83.2(75.15-84.94)	P=0.02
E/A ratio	1.06 (0.85-1.7)	1.63 (0.9-2.1)	P=0.03
PFR (ml/sec)	519.75 (409.17- 603.2)	530 (469.6-661.25)	P=ns
TPFR (m sec)	131.47 (104.58- 156.96)	134.19 (116.21- 164.31)	P=ns
NPFR	2.72 (2.31-3.34)	2.8 (2.29-3.54)	P=ns

The change in DVR_80_ and E/A ratio was not correlated with peak troponin, baseline LVEF, or baseline LV volumes.

## Conclusion

Improvement in LVEF with reperfusion did not translate to improvement in PFR, NPFR and TPFR which have been traditionally used as indices of diastolic function. On the other hand, significant changes were seen in DVR_80_ and E/A ratio. These parameters may serve as targets from future investigation in the effect of revascularization on myocardial function.

